# *Philippiphonte aspidosoma* gen. et sp. n., a radically divergent member of the Laophontidae from shell gravel in the East Sea, South Korea, including a review of *Folioquinpes* Fiers & Rutledge, 1990 (Copepoda, Harpacticoida)

**DOI:** 10.3897/zookeys.775.26404

**Published:** 2018-07-17

**Authors:** Rony Huys, Jimin Lee

**Affiliations:** 1 Department of Life Sciences, The Natural History Museum, Cromwell Road, London SW7 5BD, U.K.; 2 Marine Ecosystem and Biological Research Center, Korea Institute of Ocean Science & Technology, 385 Haeyang-ro, Yeongdo-gu, Busan, 49111, Korea

**Keywords:** Copepoda, Dokdo island, *Folioquinpes indicus* sp. n., *F. pseudomangalis* sp. n., key to species, Laophontidae, shell gravel

## Abstract

The primarily marine subtidal family Laophontidae not only contains more valid genera than any other family in the Harpacticoida, it is also one of the most speciose ones in the order, currently accommodating 327 species and subspecies. Based on published records, 25 laophontid species in 12 genera have so far been reported from Korean waters. Here both sexes of a new genus and species of Laophontidae are described, collected from shell gravel off Dokdo Island in the East Sea. *Philippiphonte aspidosoma* **gen. et sp. n.** displays a radically divergent morphology, including an extreme dorsoventrally depressed body shape which is reminiscent of members of the family Porcellidiidae. The convergent evolution of dorsoventrally flattened body plans in the Harpacticoida is briefly discussed.

The distribution and habitat preference of laophontid species recorded from the Korean peninsula are summarised. The authenticity of the Korean record of *Folioquinpes mangalis* Fiers & Rutledge, 1990 from washings of invertebrates and intertidal stones from Jeju Island is reassessed in the light of a discussion of the genus. *Folioquinpes pseudomangalis* **sp. n.** and *F. indicus* **sp. n.** are proposed as new species for *Folioquinpes mangalis* Fiers & Rutledge, 1990 *sensu* Kim (2013) and *Laophonte chathamensis* Sars, 1905 *sensu* Sewell (1924), respectively. A key to species of *Folioquinpes* Fiers & Rutledge, 1990 is provided.

## Introduction

The Laophontidae is one of the most speciose families in the Harpacticoida, currently accommodating 327 species and subspecies and containing more valid genera (74!) than any other family in the order. Members of the family can be found in tropical to polar waters and typically occur subtidally in fine to coarse-grained sandy sediments at shallow depths. Laophontids have also radiated into a wide range of other habitats, including saltmarshes and intertidal mudflats (Barnett 1968; Bodin 1976), the fronds and holdfasts of macroalgae (Hicks 1977a–b), the interstitial environment of sandy beaches (Cottarelli et al. 1986, 2008; Fiers 1990, 1991), brackish lagoons (Hamond 1972; Heip 1969; Lee and Chang 2008), anchihaline and coastal marine caves (Chappuis 1938; Huys and Lee 2000) and the deep sea (Huys and Lee 2000; Lee and Huys 1999). Some species have entered into symbiotic associations with sea anemones, bryozoans, holothurians, chitons, and particularly crustaceans (Huys 2016; Yeom et al. 2018) while a few have colonised freshwater lacustrine habitats (Defaye and Dussart 2011; Lee and Chang 2005).

Based on published records, 25 laophontid species in 12 genera have so far been reported from Korean waters. The species listed in an unpublished PhD dissertation (Kim 2002) are not considered here. *Microchelonia koreensis* (Kim, 1991) was recorded from two species of holothurian kept in aquaria at fish markets, including Holothuria (Mertensiothuria) hilla Lesson, 1830 (family Holothuriidae) in Busan (Korea Strait), and *Apostichopus japonicus* (Selenka, 1867) (family Stichopodidae) in Gangneung (East Sea coast) and Mokpo (Yellow Sea coast) (Kim 1991). The same species was subsequently collected in washings of the latter host obtained at 15 m depth in Uljin (East Sea coast) (Kim 2013). Song and Chang (1995) collected *Onychocamptus bengalensis* (Sewell, 1934) from crab burrows on an intertidal mud flat on Chindo (Jindo) Island, southwestern Korea. Lee and Chang (2005) added a second record from Sokcho, northern East Sea coast and reported another two *Onychocamptus* species [*O. mohammed* (Blanchard & Richard, 1791) and *O. vitiospinulosa* (Shen & Tai, 1963)] from streams, freshwater lakes and oligohaline reservoirs. Additional records from estuaries, lakes and saltmarshes were listed for both species by Chang (2009, 2010). *Quinquelaophonte koreana* Lee, 2003 was described from a sandy beach in Taean on the west coast of the Korean peninsula (Lee 2003) while Song et al. (2010) reported *Laophonte cornuta* Philippi, 1840, *Paralaophonte lacerdai* Jakobi, 1953, *P. obscura* Vervoort, 1962, *Heterolaophonte discophora* Willey, 1929 and *H. hamata* Jakobi, 1954 from phytal communities on *Ulva pertusa* Kjellman in Pohang, eastern Korea. Lee et al. (2012) cited *Paralaophonte congenera* (Sars, 1908) as one of the most common laophontid species in Korea. Kim (2013) listed eight (sub)species in the genus *Laophonte* Philippi, 1840 (*L. cornuta*; *L. thoracica* Boeck, 1865; *L. inopinata* T. Scott, 1892; *L. denticornis*, T. Scott, 1894; *L. inornata* A. Scott, 1902; *L. dinocerata* Monard, 1926; *L. elongata barbata* Lang, 1934; *L. longistylata* Willey, 1935), three species in the genus *Paralaophonte* Lang, 1948 [*P. macera* (Sars, 1908), *P. lacerdai*, *P. obscura*], in addition to *Heterolaophonte discophora*, *Harrietella simulans* T. Scott, 1906, *Echinolaophonte mirabilis* (Gurney, 1927), *Folioquinpes mangalis* Fiers & Rutledge, 1990, *Robustunguis minor* Fiers, 1992 and *Psammoplatypus proprius* (Lang, 1965). Several of these species were found in submerged wood infested by teredinid shipworms and limnoriid isopods or in washings of invertebrates such as sponges, barnacles, soft corals, and oysters. However, the great majority of these records should be considered as accidental associations (Huys 2016). Finally, *Jejulaophonte hyeopjaeensis* Back & Lee, 2014 was recently described from a sandy beach on Jeju Island (Back and Lee 2014).

Both sexes of a new species were collected from shell gravel off Dokdo Island in the East Sea. The new species displays a radically divergent morphology and cannot be accommodated in any of the currently recognised genera. It is here fixed as the type species of a new genus, *Philippiphonte* gen. n., and described in detail. The authenticity of the Korean record of *Folioquinpes mangalis* from washings of invertebrates and intertidal stones from Jeju Island is reassessed in the light of a review of the genus *Folioquinpes* Fiers & Rutledge, 1990.

## Materials and methods

**Figure 1.**
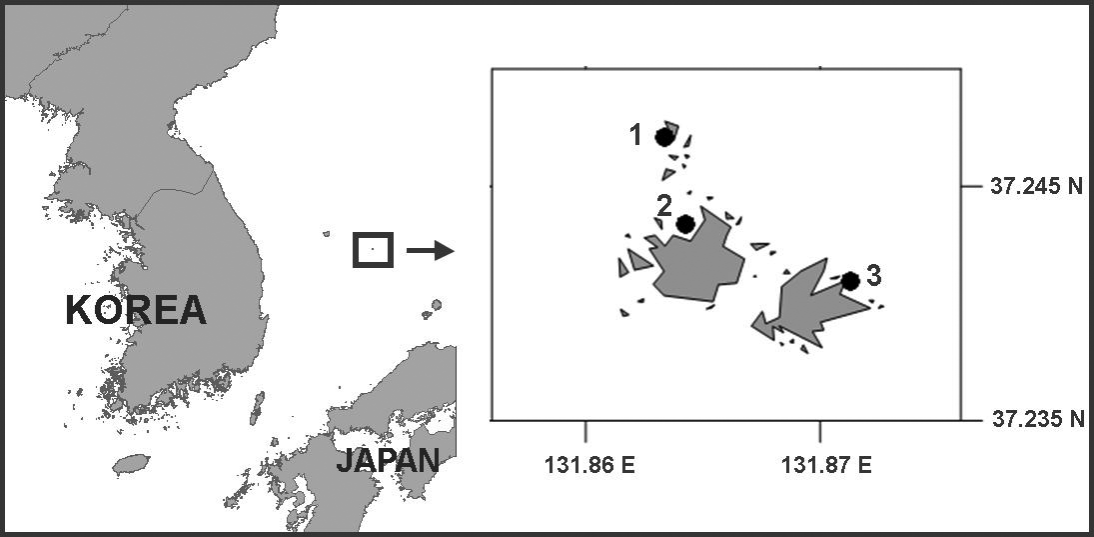
Localities in Dokdo island, Korea where *Philippiphonte aspidosoma* gen. et sp. n. was collected: **1** Gajaebawi (type locality) **2** Mulgol **3** Old harbour.

Samples were collected by SCUBA diving by scooping the upper ~ 5 cm of sublittoral sediments around Dokdo Island, East Sea (Sea of Japan), South Korea (Figure 1) during April and June 2015, and August 2016, and transferred to 1-litre plastic bottles to which 7% MgCl_2_ solution was added; after 5–10 min the samples were fixed in 10% formalin. In the laboratory, specimens were extracted from the sediments by flotation-centrifugation using the Ludox HS-40 colloidal silica polymer (Burgess 2001), and rinsed and filtered through a 63 μm mesh-size sieve with tap water. Copepods were sorted under a Leica M165C stereomicroscope, transferred to glycerine, and then dissected in lactic acid. Whole specimens and appendages were drawn using a camera lucida mounted on a Leica DM2500 microscope equipped with differential interference contrast. After examination, the dissected parts were mounted in lactophenol mounting medium and sealed.

The descriptive terminology is adopted from Huys and Boxshall (1991) and Huys et al. (1996). Abbreviations used in the text are: ae, aesthetasc; P1–P6, for swimming legs 1–6; exp, enp and benp for exopod, endopod and baseoendopod, respectively; exp (enp)-1 (-2, -3) to denote the proximal (middle, distal) segments of a ramus; apo for apophysis. The term ‘acrothek’ denotes the trifid setal structure found primitively on the apical margin of the distal antennulary segment (Huys and Iliffe 1998).

Type specimens were deposited in the National Biological Resources Center (NIBR), Incheon, Republic of Korea. Additional material was stored in the Korea Institute of Ocean Science and Technology (KIOST), Busan, Korea.

## Systematics

### Order Harpacticoida Sars, 1903

#### Family Laophontidae T. Scott, 1905

##### Subfamily Laophontinae T. Scott, 1905 *sensu* Huys & Lee (2000)

###### Genus Philippiphonte gen. n.

Taxon classificationAnimaliaHarpacticoidaLaophontidae

http://zoobank.org/F82FCADE-CF6B-4AAF-A465-F9DC8E73F9CD

####### Diagnosis.

Laophontidae. Body extremely dorsoventrally flattened, porcellidiid-like. Distinct sexual dimorphism in size, urosomal segmentation, antennule, P3 endopod, P5, and P6. Rostrum large, inverted trapezoid; anterior margin slightly convex in ♀, virtually straight in ♂. Cephalothoracic shield broadly bell-shaped; lateral margins fringed with closely set spinules. Pedigerous somites bearing legs 2–4 with strongly developed pleurotergites, those of leg 4-bearing somite backwardly produced and embracing leg 5-bearing somite and anterior half of genital double-somite; each provided with strong spinules along lateral margins. Leg 5-bearing somite reduced, without marked pleurotergites. Genital double-somite completely fused. Second and third abdominal somites with lobate pleurotergites, those of penultimate somite embracing anal somite and anterior half of caudal rami. Anal somite without expanded pleurotergites; operculum naked. Caudal rami flattened, longer than wide, with straight outer and markedly convex inner margin; with medially directed spinules along inner margin and finer spinules along outer margin; with seven setae, all of which located near posterior margin of ramus; setae IV–V with fracture planes and fused at base.

Antennule slender, 5-segmented and with aesthetasc on segment 3 in ♀, subchirocerate, 8-segmented and with aesthetasc on segment 5 in ♂; without spinous processes on segments 1–2; segments 1–3 with setules along anterior margin; segment 3 elongate in ♀. Antenna with allobasis bearing unipinnate seta along abexopodal margin. Exopod 1-segmented, with four elements. Mandible with slender gnathobase; palp small, comprising unisetose basis with incorporated endopod and discrete exopod, armed with three and one seta(e), respectively. Maxillule without defined rami; armature of palp represented by one lateral and three distal setae. Maxilla with two coxal endites; endopod with two setae. Maxilliped elongate and slender; syncoxa with one seta; endopod represented by acutely recurved claw with minute accessory seta at its base.

Legs 1–4 with very wide and narrow intercoxal sclerites. Leg 1 with sparsely plumose inner and outer seta on basis; exopod 3-segmented with long outer spine on exp-1, extending beyond distal margin of exp-3 and bearing stiff spinules along its outer margin; exp-2 with outer unipinnate spine; exp-3 with two unipinnate spines and two geniculate setae; endopod 2-segmented, prehensile, enp-1 unarmed, enp-2 with short claw but accessory seta not discernible. Legs 2–4 with transversally elongate bases, with long (P2–P3) or short (P4) outer seta; with 3-segmented exopods and 2-segmented endopods (except for P3 endopod 3-segmented in ♂); outer exopodal spines typically unipinnate in distal half only, inner setae very long and plumose; outer margin of P2–P4 enp-2 with double row of flimsy setular extensions. Leg 3 ♂ with outwardly recurved, spinous apophysis on enp-2; enp-3 with one inner and two apical setae. Armature formulae:

**Table:** 

	**Exopod**	**Endopod**
P2	0.1.123	0.120
P3	0.1.223	0.121 [0.apo.120 in ♂]
P4	0.1.223	0.121

Leg 5 biramous; baseoendopod very elongate, backwardly recurved, with outer basal seta arising from short dorsal setophore; endopodal armature represented by three setae in ♀ and one seta in ♂; exopod with four elements in ♀ and three elements in ♂.

Genital field ♀ located near border with leg 5-bearing somite. P6 forming well developed operculum with two small setae in ♀; asymmetrical in ♂ (with dextral or sinistral configuration), with outer distal corner bearing one minute seta.

**Type species.** *Philippiphonte aspidosoma* gen. et sp. n. (by original designation).

**Etymology.** The genus is dedicated to Rudolph Amandus Philippi (14 September 1808–23 July 1904), author of the type genus *Laophonte* Philippi, 1840 and of the first publication to adopt the term “copepod” in its title (Philippi 1843). Many of Philippi’s (1840, 1843) generic names such as *Aenippe*, *Euryte*, *Idomene*, *Idya* (= *Tisbe*), *Metis*, *Oncaea*, *Psamathe* (= *Scutellidium*) and *Thyone* (= *Porcellidium*) were named after figures of Ancient Greek mythology and so was also *Laophonte*, named after a daughter of Pleuron, and the wife of Thestius, by whom she had Althaea and Leda.

###### Philippiphonte aspidosoma sp. n.

Taxon classificationAnimaliaHarpacticoidaLaophontidae

http://zoobank.org/7C22C6B6-91CD-42FC-934E-4FCCEAA5979B

**Figure 2.**
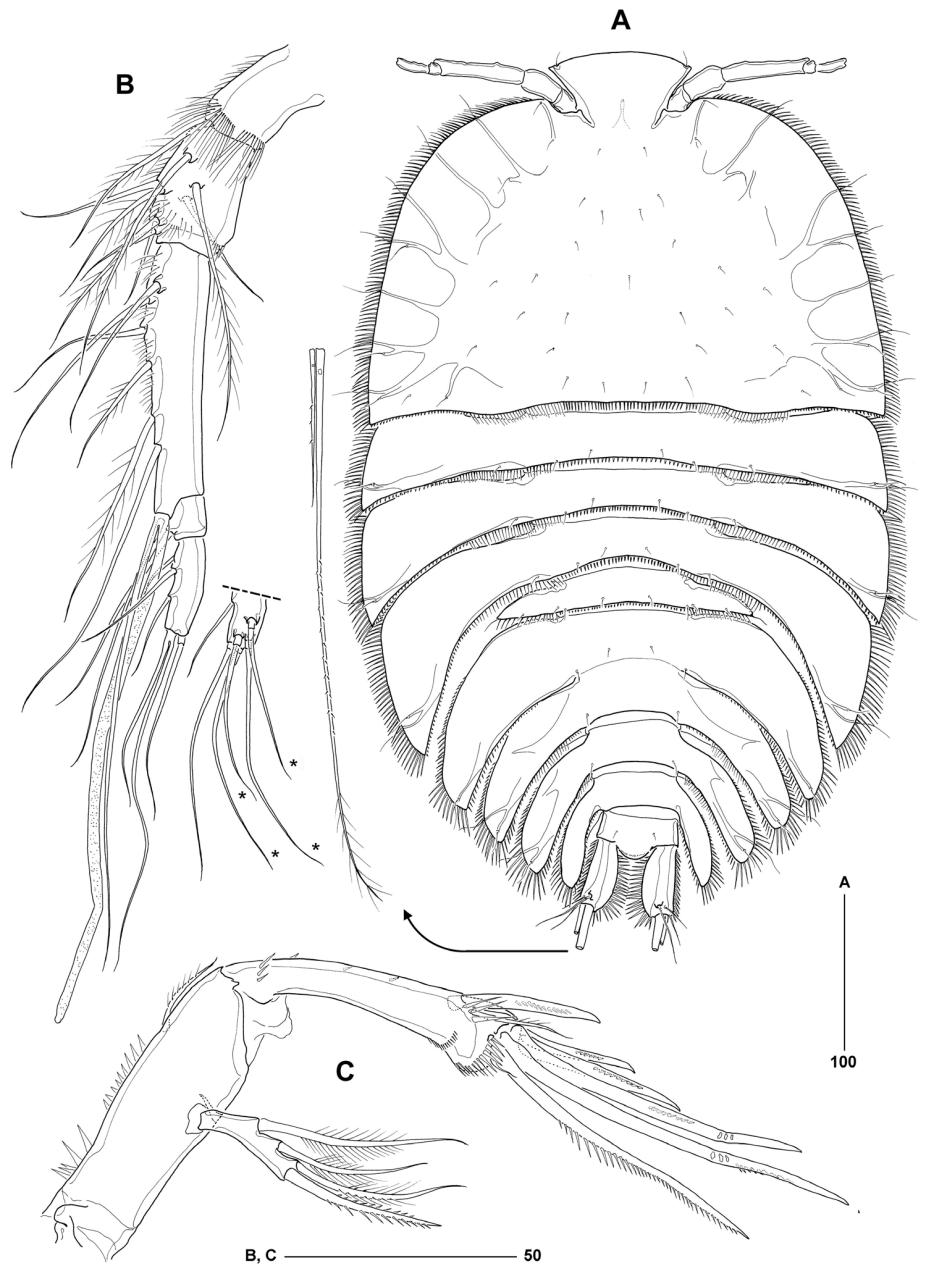
*Philippiphonte aspidosoma* gen. et sp. n. (♀): **A** habitus, dorsal [inset showing caudal ramus setae IV–V at full length] **B** antennule, ventral [inset showing apical armature of segment 5 in dorsal aspect; dorsal setae not shown in ventral aspect marked by *] **C** antenna.

**Figure 3.**
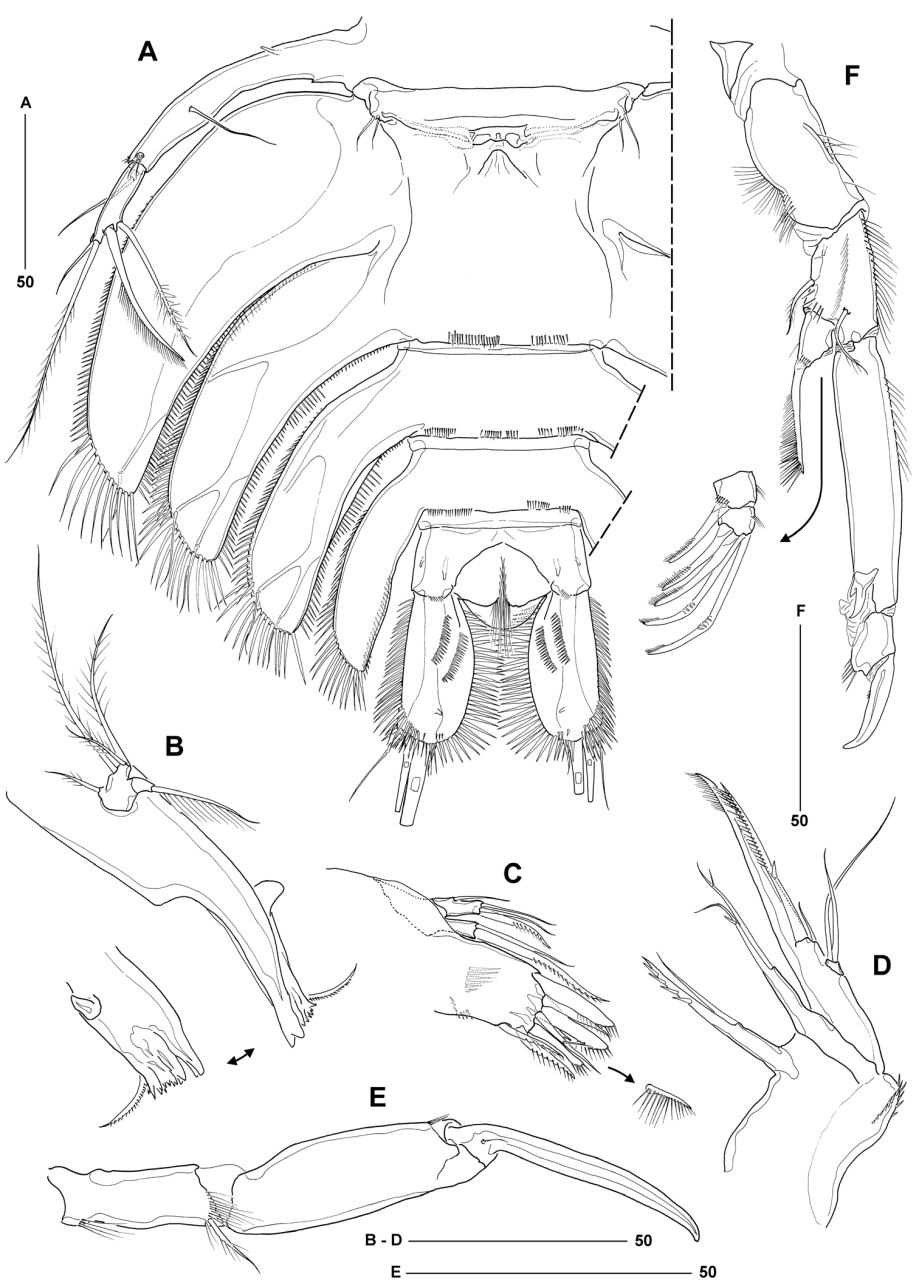
*Philippiphonte aspidosoma* gen. et sp. n. (♀): **A** urosome and right leg 5, ventral **B** mandible [inset showing gnathobase from different angle] **C** maxillule, anterior [inset showing small unipinnate element arising from posterior surface] **D** maxilla **E** maxilliped **F** leg 1, anterior [exp-2 and -3 disarticulated].

**Figure 4.**
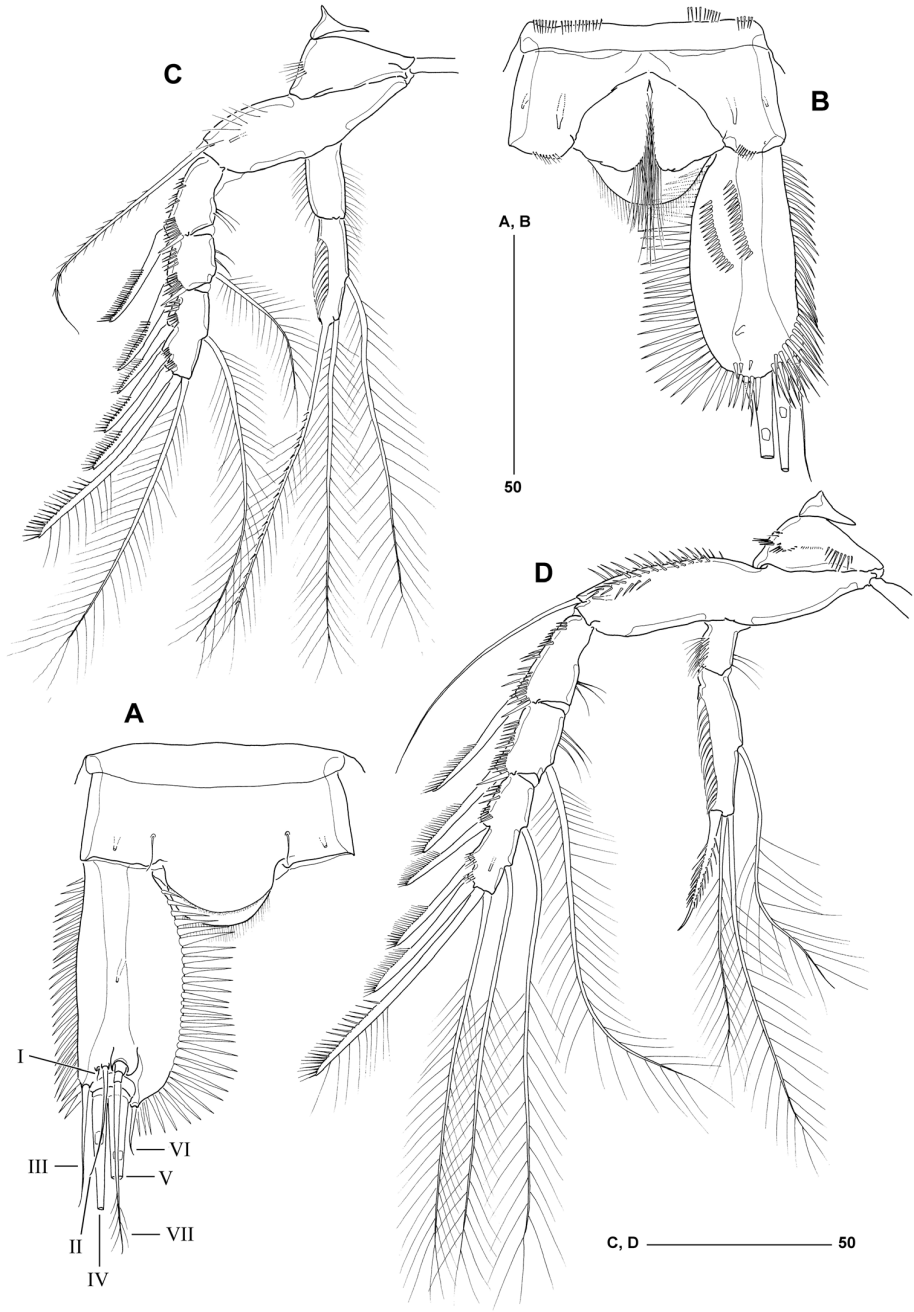
*Philippiphonte aspidosoma* gen. et sp. n. (♀): **A** anal somite and left caudal ramus, dorsal **B** anal somite and left caudal ramus, ventral **C** leg 2, anterior **D** leg 3, anterior.

**Figure 5.**
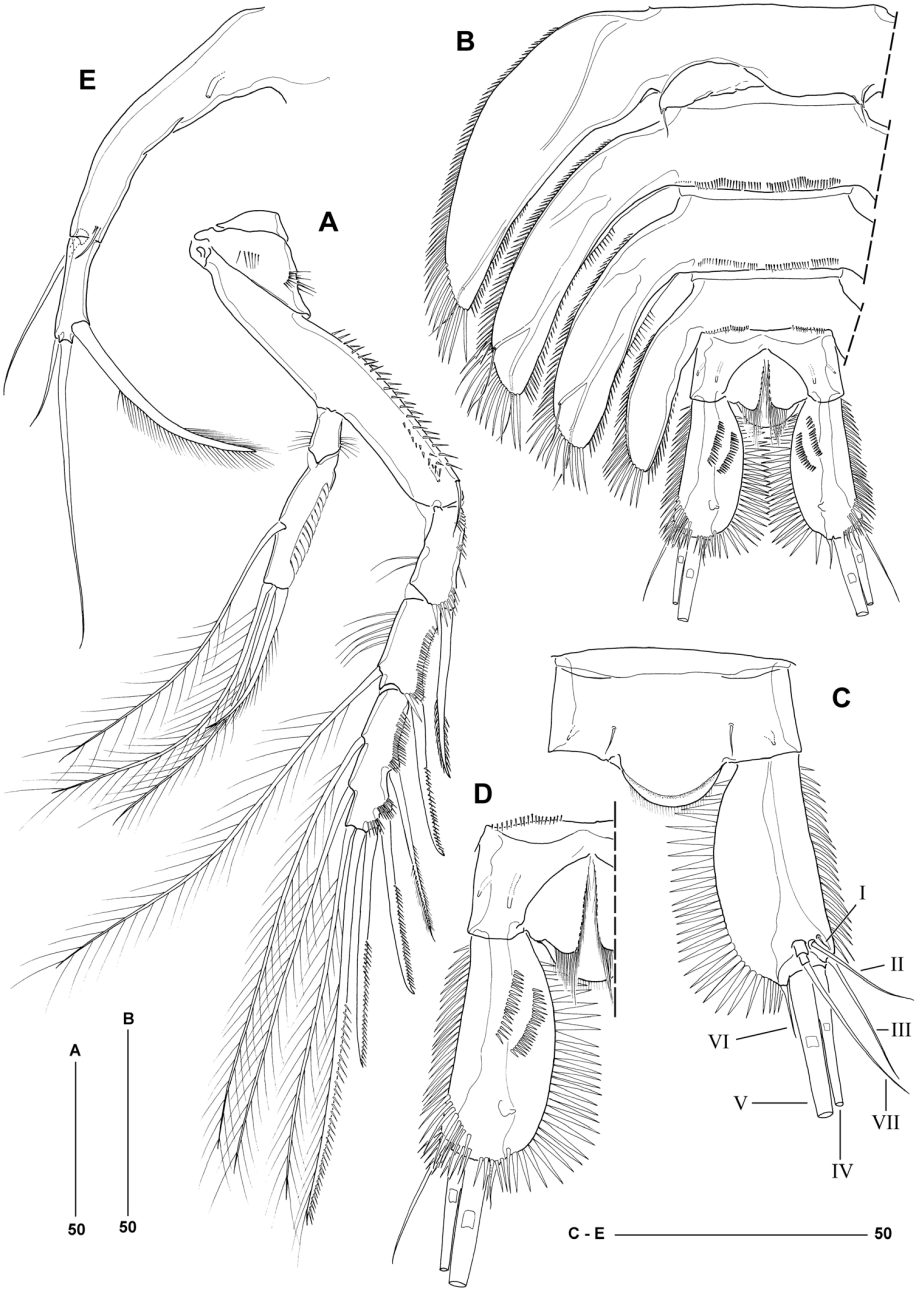
*Philippiphonte aspidosoma* gen. et sp. n.: **A** leg 4 ♀, anterior **B** urosome ♂ (excluding leg 5-bearing somite), ventral **C** anal somite and right caudal ramus ♂, dorsal **D** anal somite and right caudal ramus ♂, ventral **E** leg 5 ♂, ventral.

**Figure 6.**
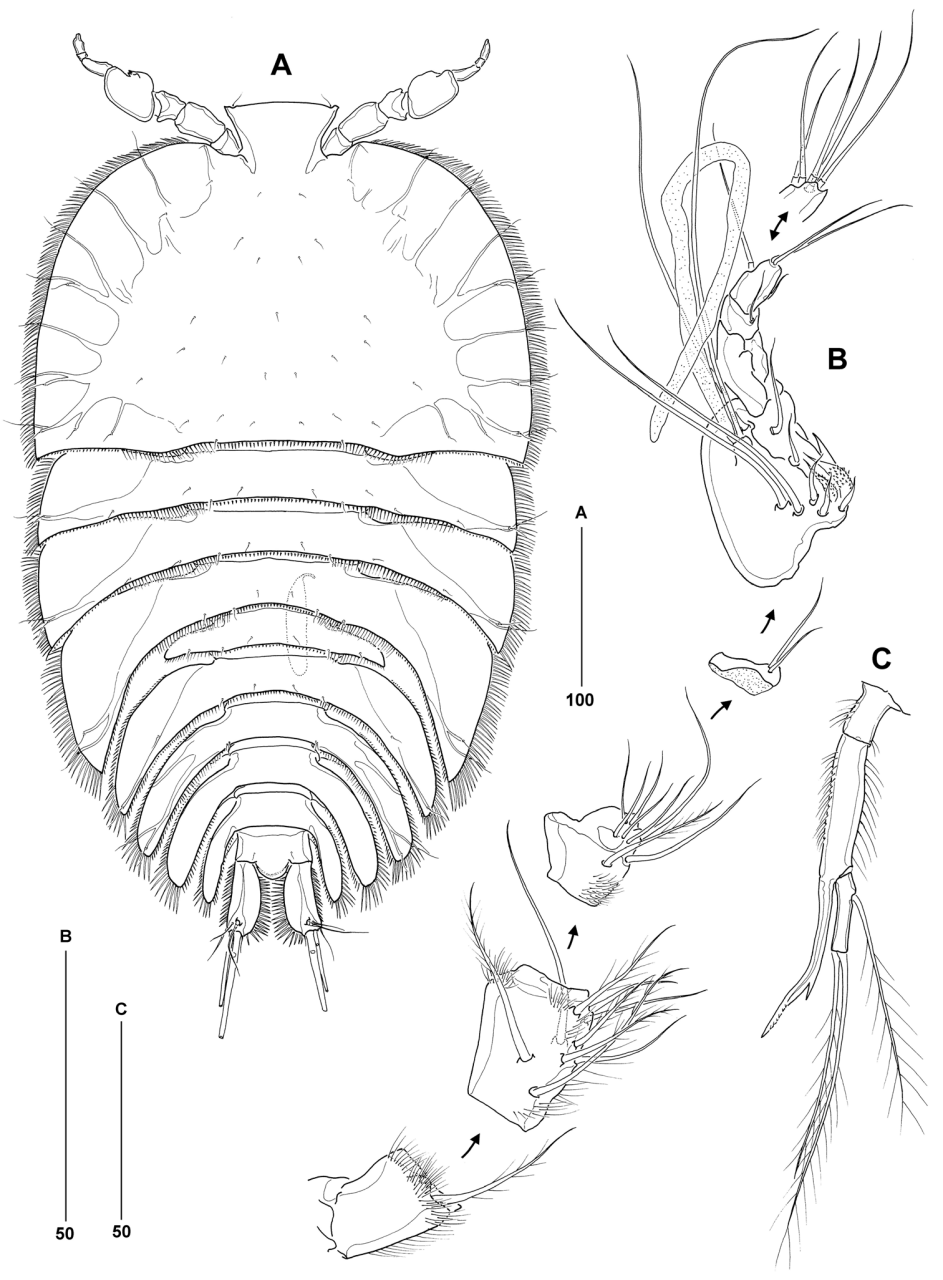
*Philippiphonte aspidosoma* gen. et sp. n. (♂): **A** habitus, dorsal **B** antennule, ventral [segments 1–4 disarticulated; insert showing apical armature of segment 8 in dorsal aspect] **C** leg 3 endopod, anterior.

**Figure 7.**
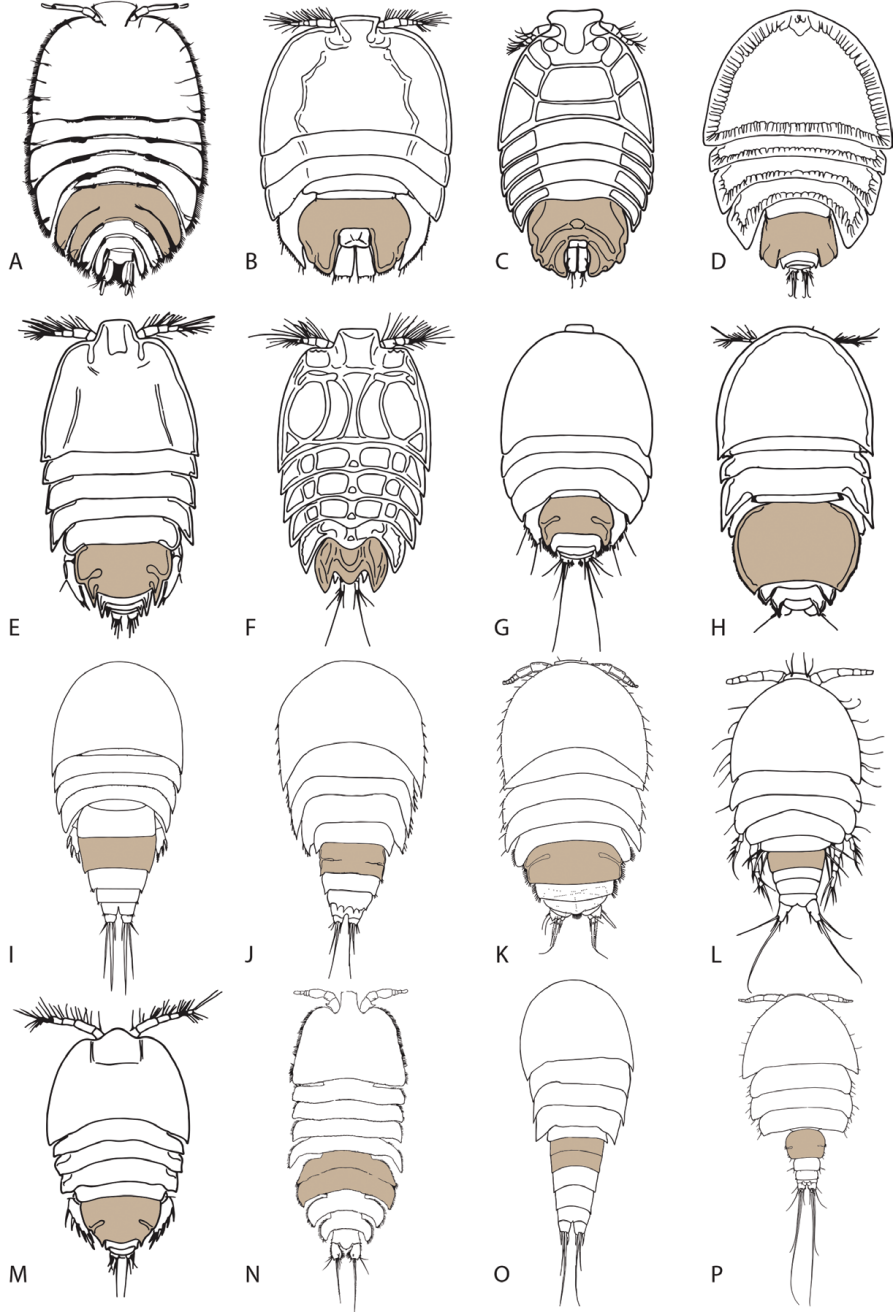
Harpacticoid copepods exhibiting dorsoventrally flattened body shapes (females only, dorsal view; genital double-somite shaded). **A** *Philippiphonte aspidosoma* (Laophontidae) **B** *Porcellidium viride* (Philippi, 1840) (Porcellidiidae) **C** *Neopeltopsis pectinipes* Hicks, 1976 (Peltidiidae) **D** *Hamondia superba* Huys, 1990 (Hamondiidae) **E** *Alteutha oblonga* (Goodsir, 1845) (Peltidiidae) **F** *Peltidium purpureum* Philippi, 1839 (Peltidiidae) **G** *Zaus abbreviatus* Sars, 1904 (Harpacticidae) **H** *Paramenophia platysoma* (Thompson & Scott, 1903) (Thalestridae) **I** *Xouthous purpurocinctus* (Norman & Scott, 1905) (Pseudotachidiidae) **J** *Xouthous parasimulans* (Médioni & Soyer, 1968) (Pseudotachidiidae) **K** *Mucropedia kirstenae* Bouck, Thistle & Huys, 1999 (Harpacticidae) **L** *Donsiella phycolimnoriae* Hicks, 1990 (Pseudotachidiidae) **M** *Alteuthoides kootare* Hicks, 1986 (Peltidiidae) **N** *Peltidiphonte rostrata* Gheerardyn & Fiers in Gheerardyn, Fiers, Vincx & De Troch, 2006 (Laophontidae) **O** *Peltobradya bryozoophila* Médioni & Soyer, 1968 (Ectinosomatidae) **P** *Scutellidium arthuri* Poppe, 1884 (Tisbidae).

Figs 2 , 3 , 4 , 5 , 6 , 7

####### Type locality.

South Korea, East Sea (Sea of Japan), Gajaebawi, Dokdo island (Liancourt Rocks), 37°14'49.37"N, 131°51'48.24"E, shell gravel, 22 m depth (Figure 1).

**Type material.** Holotype ♀ dissected on 11 slides (reg. no NIBRIV0000816435), allotype ♂ dissected on 11 slides (reg. no NIBRIV0000816434), remaining paratypes (9 ♀♀, 1 ♂) preserved in formalin (reg. no NIBRIV0000816433). All type specimens were collected on 23 April 2015 from the type locality and are deposited in the National Biological Resources Center (NIBR), Incheon.

####### Additional material examined.

1 ♂ from Mulgol, Dokdo island, 37°14'35.16"N, 131°51'51.37"E, 15 m depth, 27 June 2015 (reg. no MInRB-Hr15-L001); 1 ♂ from the old harbour of Dokdo island, 37°14'27.31"N, 131°52'16.69"E, 12 m depth, 27 June 2015 (reg. no MInRB-Hr15-L002); 2 ♀♀, 4 ♂♂, 24 August 2016 from type locality (Figure 1) (reg. no MInRB-Hr15-L003). All specimens are deposited in the collections of the Korea Institute of Ocean Science and Technology (KIOST), Busan.

####### Description of female.

Body length from anterior margin of rostrum to posterior margin of caudal rami 536–612 μm (mean = 574 μm; *n* = 12; holotype = 552 μm); maximum width measured at level of leg 3-bearing somite: 338 μm (in holotype). Body (Figure 2A) extremely dorsoventrally flattened, porcellidiid-like; except for digestive tract and ovaries completely transparent; dorsal surface of all somites covered with minute setules and denticles (not illustrated); ventral surface of urosomites without surface ornamentation (except for spinule rows around posterior margin). Rostrum large, prominent in dorsal aspect, inverted trapezoid; anterior margin slightly convex, anterolateral corners each with sensillum. Cephalothoracic shield broadly bell-shaped, about 1.5 times wider than long; lateral margins fringed with closely set spinules; dorsal surface with symmetrical pattern of sensilla; posterior margin with setules and spinules. Pedigerous somites bearing legs 2–4 with strongly developed pleurotergites, those of leg 4-bearing somite backwardly produced and embracing leg 5-bearing somite and anterior half of genital double-somite; each provided with strong spinules along lateral margins and shorter spinules along posterior margin; sensillar pattern as illustrated. Leg 5-bearing somite reduced, without marked pleurotergites; posterior margin with spinules dorsally and setules dorsolaterally. Genital double-somite completely fused; original segmentation marked by sensillar pattern, faint dorsal suture and paired arrangement of backwardly directed pleurotergites, each fringed with long spinules laterally and shorter spinules or setules posteriorly; anterior pair larger than posterior pair; ventral posterior margin with paired rows of tiny spinules (Figure 3A). Second and third abdominal somites with lobate pleurotergites, those of penultimate somite embracing anal somite and anterior half of caudal rami; dorsal and lateral ornamentation as in previous somites; ventral posterior margin with paired rows of tiny spinules (Figure 3A). Anal somite without expanded pleurotergites (Figs 2A; 4A–B); dorsal surface with paired tube-pores and sensilla flanking rounded, naked anal operculum; ventral surface with two pairs of tube-pores and tiny spinules near bases of caudal rami; anal frill triradiate, well developed, provided with long setular extensions.

Caudal rami (Figure 4A–B) flattened, about 2.2 times longer than maximum width, with straight outer and markedly convex inner margin; with elaborate ornamentation consisting of strong, medially directed spinules along inner margin, finer spinules along outer margin, and two spinule rows in anterior half of ventral surface. Armature consisting of seven setae, all of which located near posterior margin of ramus; seta I minute, positioned dorsally near naked seta II; seta III located at outer distal corner, naked; setae IV–V with fracture planes and fused at base; seta IV sparsely pinnate, about 18% of body length; seta V very long, about 3.5 times length of seta IV (Figure 2A), with minute spinules in middle third and sparse setules in distal quarter; seta VI located at inner distal corner; seta VII located near posterior margin of ramus, tri-articulate at base and sparsely plumose in distal third.

Antennule (Figure 2B) 5-segmented, slender; without spinous processes on segments 1–2; segment 1 with setules along anterior and ventral distal margin, those on the latter being particularly long; anterior margin of segments 2 and 3 (proximal half only) with short setules; segment 3 longest, about 2.4 times as long as segment 1 (measured along anterior margin), with aesthetasc (114 μm) arising from socle and fused at base to long naked seta. Armature formula 1-[1 plumose], 2-[4 + 4 plumose], 3-[5 + 2 plumose + (1 + ae)], 4-[1], 5-[8 + acrothek]; apical acrothek consisting of two basally fused setae, aesthetasc not observed.

Antenna (Figure 2C) with allobasis, bearing two spinule rows and slender unipinnate seta along abexopodal margin. Exopod 1-segmented, with two lateral and two apical bipinnate setae (outer one slightly spiniform). Free endopod with two spines and one seta laterally, and distal armature consisting of two geniculate setae, one long (fused at base to vestigial seta) and two short pinnate spines.

Mandible (Figure 3B) with slender gnathobase bearing several multicuspidate teeth and one unipinnate seta. Palp small, comprising basis with incorporated rami; armature of basis represented by one plumose seta originating from small articulating socle; endopod represented by one short and two long plumose setae; exopod represented by one sparsely pinnate seta.

Maxillule (Figure 3C) with well-developed syncoxal arthrite bearing two spinule rows on posterior surface and total of eight elements along distal margin. Coxal endite with one naked seta and one unipinnate spine. Basis without defined rami; armature represented by one lateral and three distal setae (innermost of which spiniform and unipinnate).

Maxilla (Figure 3D). Syncoxa with spinules along distal outer margin and two coxal endites; proximal endite with naked seta and basally fused unipinnate spine, distal endite with two setae of which innermost one fused at base. Allobasis produced into distally unipinnate claw, with accessory armature consisting of small naked seta and unipinnate spiniform element. Endopod represented by a minute segment with two basally fused setae.

Maxilliped (Figure 3E) elongate and slender. Syncoxa with one sparsely plumose seta and tuft of long setules near distal inner corner and additional inner setules around base. Basis without ornamentation except for few spinules near outer distal corner. Endopod represented by acutely recurved claw with minute accessory seta at its base.

Leg 1 (Figure 3F) with very wide and narrow intercoxal sclerite. Basis with sparsely plumose inner (anterior) and outer seta. Exopod 3-segmented, all segments of about equal size; exp-1 with long outer spine, extending beyond distal margin of exp-3 and bearing stiff spinules (gradually increasing in size distally) along its outer margin; exp-2 and -3 wider than long, with tuft of setules along inner margin; exp-2 with outer spine being unipinnate in its distal half; exp-3 with two unipinnate spines and two geniculate setae (pinnules restricted to apical parts of elements). Endopod 2-segmented, prehensile; enp-1 elongate, about five times as long as wide, unarmed, with long spinules along proximal half of inner margin; enp-2 with short, acutely recurved claw, outer distal corner with few spinules but accessory seta not discernible.

Legs 2–4 (P2–P4) (Figs 4C–D; 5A) with widely separated members connected by narrow intercoxal sclerites. Praecoxae represented by small U-shaped sclerite. Coxae with spinular ornamentation on anterior surface as figured. Bases transversally elongate, becoming progressively longer from P2 to P4; outer margin with setules (P2) or multiple rows of spinules (P3–P4); with long (P2–P3) or short (P4) outer seta, bipinnate in P2 only; anterior surface with tube-pore. Exopods 3-segmented; exp-1 without inner seta; inner margin of exp-1 and -2 with few long setules; outer margin of all segments with spinular ornamentation as figured; P3 exp-3 with tube-pore on anterior surface; outer exopodal spines typically unipinnate in distal half only (except for outer spine of exp-1 and proximal outer spine on exp-3 of P4 being bipinnate); inner setae very long and plumose. Endopods 2-segmented; enp-1 unarmed, shorter than enp-2, with setules along both inner and outer margins; outer margin of enp-2 with double row of flimsy setular extensions; outer distal spine of P3 enp-2 bipinnate. Spine and setal formulae of swimming legs as for genus.

Leg 5 (Figure 3A) consisting of baseoendopod and 1-segmented exopod. Baseoendopod subcylindrical and elongate (about 8.5 times as long as average width), backwardly recurved and fused at base to pleural wall of somite; bearing outer basal seta arising from short setophore (located dorsally); endopodal armature consisting of long seta located at about two-thirds the segment length, and two closely set, minute setae originating near boundary with exopod; all setae naked; proximal third with tube-pore on ventral surface. Exopod about one third the size of baseoendopod; inner margin with one bipinnate and one unipinnate seta, distal margin with long plumose and short naked seta.

Genital field (Figure 3A) located in anterior third of genital double-somite, near border with leg 5-bearing somite. Genital apertures closed off by opercula derived from vestigial sixth legs, each bearing two minute, naked setae. Copulatory pore median, of moderate size. Egg-sac not observed.

####### Description of male.

Slightly smaller than female; body length from anterior margin of rostrum to posterior margin of caudal rami 461–527 μm (mean = 489 μm; *n* = 8; allotype = 523 μm); maximum width measured near posterior margin of cephalothorax: 315 μm (in allotype). Body (Figure 6A) of similar shape, transparency and with virtually identical ornamentation as in female. Rostrum comparatively narrower than in female and with virtually straight anterior margin. Genital and first abdominal somites completely free; posterior margin of former with continuous row of short spinules or setules posteriorly; lobate pleurotergites of genital somite more slender than in female. Anal somite and caudal rami (Figure 5B–D) as in female.

Antennule (Figure 6B) 8-segmented, subchirocerate, with geniculation between segments 5 and 6; without spinous processes on segments 1–2. Segment 1 as in female; anterior margin of segments 2 and 3 (proximal half only) with setules; segment 4 represented by an incomplete U-shaped sclerite; segment 5 swollen, with large aesthetasc (125 μm) arising from socle and fused at base to long naked seta; segments 5 and 6 with setae modified into basally fused spinous processes. Armature formula 1-[1 plumose], 2-[4 + 5 plumose], 3-[7 + 1 plumose], 4-[2], 5-[8 + 2 spinulose + 2 spinous processes + (1 + ae)], 6-[2 spinous processes], 7-[1], 8-[7 + acrothek]; apical acrothek consisting of two basally fused setae, aesthetasc not observed.

Leg 3 (Figure 6C) with 3-segmented endopod. Enp-1 shortest, with few setules on both outer and inner margins. Enp-2 forming slender, outwardly recurved, spinous apophysis (homologue of outer distal spine of enp-2 in female) provided with barb along inner margin and minute projections near apex; setules present on both outer and inner margins. Enp-3 with one inner and two apical plumose setae.

Leg 5 (Figure 5E) consisting of baseoendopod and 1-segmented exopod. Baseoendopod elongate, backwardly recurved and fused to pleural wall of somite; bearing outer basal seta arising from short setophore (located dorsally); endopodal armature consisting of two closely set, vestigial setae near boundary with exopod; proximal third with tube-pore on ventral surface. Exopod about one third the size of baseoendopod; inner margin with one strong, bipinnate seta, distal margin with one long and one short naked seta.

Sixth legs (P6) (Figure 5B) asymmetrical with functional right member articulating at base and closing off genital aperture and left member fused at base to genital somite; each vestigial sixth leg with minute naked seta. Spermatophore oval, relatively small (65 μm).

####### Etymology.

The specific epithet is derived from the Greek άσπίς, meaning shield, and σῶμα, meaning body, and alludes to the dorsoventrally flattened shield-shaped body form.

#### Discussion

##### Taxonomic position of *Philippiphonte aspidosoma* gen. et sp. n.

The new genus can readily be identified as a member of the family Laophontidae because of the morphology of leg 1, including the presence of a pedestal on the basis used for the insertion of the endopod, the displacement of the inner spine onto the anterior surface of the basis, and the modification of the outer (= anterior) distal element of enp-2 into a large non-geniculate claw (Huys 1990a). The inner (= posterior) distal element on enp-2 which is typically reduced to the size of a setule in laophontids appears to be absent in *P. aspidosoma*. The new genus is placed in the subfamily Laophontinae based on the following synapomorphies as defined by Huys and Lee (2000): (a) male antennule with up to three segments distal to geniculation, (b) mandible without discrete exopod, (c) maxilliped with maximum two setae on syncoxa, (d) P1 enp-1 without inner seta, (e) P2 enp-2 without outer spine, (f) proximal outer setae of female P5 exopod with distinctly separated insertion sites, and (g) absence of cup-shaped transformed pores on legs, somites or caudal rami.

*Philippiphonte aspidosoma* is morphologically radically divergent from other members of the family, justifying its assignment to a new genus. Unique autapomorphies that define the genus *Philippiphonte* include (a) the extremely dorsoventrally flattened, porcellidiid-like body shape in both sexes (Figs 2A, 6A), (b) the inverted trapezoid shape of the rostrum (Figs 2A, 6A), (c) the flattened caudal rami with elaborate spinular ornamentation along inner and outer margins (Figs 4A–B, 5C–D), (d) the remarkably slender antennules in the ♀, characterised by a very elongate third segment (Figure 2B), (e) basal seta of mandibular palp originating from small articulating socle (Figure 3B), (f) P1 exp-1 with long outer spine, extending beyond distal margin of exp-3 and bearing stiff spinules (Figure 3F), (g) legs 2–4 with widely separated members connected by narrow intercoxal sclerites and with transversally elongate bases, becoming progressively longer from P2 to P4 (Figs 4C–D, 5A), and (h) P5 baseoendopod subcylindrical, elongate, backwardly recurved and fused at base to pleural wall of supporting somite in both sexes (Figs 3A, 5E). Another character of interest is the presence of only one seta on the male sixth legs (Figure 5B), the plesiomorphic 2-setae condition being typical for members of the Laophontidae. The only other reported exception is found in the esolinid *Applanola hirsuta* (Thompson & Scott, 1903) which has unarmed sixth legs (Huys and Lee 2000: fig. 19D).

The interrelationships of the Laophontidae are poorly resolved despite decades of morphological studies with the least confidently resolved part of the tree being the relative positions of the 65 genera in the subfamily Laophontinae. The significance of patterns of swimming leg sexual dimorphism in unravelling relationships among certain laophontid lineages has been demonstrated repeatedly in a number of studies (e.g., Lee and Huys 1999; Gómez and Boyko 2006; McCormack 2006; Huys and Lee 2009). However, except for the apophysis on the male P3 endopod (Figure 6C) no other sexual dimorphism is expressed on legs 2–4 of *P. aspidosoma*. The presence of such an apophysis is phylogenetically uninformative at generic level since it is a convincing synapomorphy uniting the families of the Laophontoidea (Huys 1990a; Huys and Lee 1998, 1999). Given the many autapomorphic character traits expressed in its body plan, the identity of the closest relative of *P. aspidosoma* will probably remain elusive until the arrival of molecular data. The swimming legs of *P. aspidosoma* are characterised by the presence of a double row of setular extensions along the weakly chitinised outer margin of P2–P4 enp-2 (Figs 4C–D, 5A). Similar parallel rows of flimsy extensions, possibly surrounding a glandular opening, have previously been reported in *Marbefia carthyi* (Hamond, 1968) (Huys and Lee 2009: Figs 5B, 6A–C). Although the latter represent positional homologues to the structures observed in *P. aspidosoma* there is no additional morphological evidence suggesting a close relationship between *Philippiphonte* and *Marbefia* Huys & Lee, 2009. The same applies to the laophontid genera *Asellopsis* Brady & Robertson, 1873, *Platylaophonte* Bodin, 1968, *Applanola* Huys & Lee, 2000 and *Peltidiphonte* Gheerardyn & Fiers in Gheerardyn et al., 2006, all of which have a more or less dorsoventrally depressed body shape, but display no other apomorphic similarities in support of a direct relationship with *Philippiphonte*. As previously suggested by Gheerardyn et al. (2006) the somewhat similar body shape in these genera is more than likely the result of convergent evolution.

All members of the families Porcellidiidae and Peltidiidae are exclusively dorsoventrally depressed. However, flattened body shapes have also evolved in many other harpacticoid lineages (Figure 7). In some harpacticoid genera the dorsoventral flattening of the body is an adaptation to a mode of life associated with smooth, flat surfaces such as macroalgae (Noodt 1971; Hicks 1980) or the inside surface of gastropod shells used by anomuran decapods (Huys 2016). Since such substrates are commonly exposed to strong water currents, a low-profile body shape evidently helps the copepod maintaining its position on the surface, designed to disturb the water flow as little as possible. In other cases, dorsoventral flattening has been assumed to be an adaptation to life on coral fragments in an environment with strong currents (Gheerardyn et al. 2006) or with invertebrate hosts (Huys 1990b). In its least modified form flattening involves only the prosome with no (e.g., *Scutellidium* spp.; Figure 7P) or very moderate (e.g., *Xouthous* spp.; *Donsiella* spp.; *Peltobradya* spp.; Figure 7I–J, L, O) dorsoventral depression of the urosome. However, in most low-profile body shapes the latter tagma displays a similar modification as the prosome, often blurring the boundary between both. A common way by which urosomal flattening is achieved is by enlargement of the genital double-somite resulting from transversal expansion (Figure 7G–H, K, M) and/or the formation of pleural wings (Figure 7A–D, F, N). The genital double-somite can become very large in relation to the rest of the body (e.g., *Paramenophia* spp.; Figure 7H) and can incorporate additional somites posterior to it to form a genital complex which embraces the anal somite and caudal rami. This condition is found in some genera of the Peltidiidae (*Neopeltopsis* Hick, 1976; Figure 7C, F) and all members of the Porcellidiidae (Figure 7B).

The shape of the rostrum, the general ovoid, shield-shaped habitus and the degree of dorsoventral compression in *Philippiphonte* are somewhat reminiscent of the condition in the Porcellidiidae (compare Figure 7A, B). However, the morphology of the rest of the body shows important differences. In all Porcellidiidae the dorsal cephalic shield and epimeral plates of the free pedigerous somites are typically provided with a marginal hyaline membrane. Hence, during attachment the body is sealed around most of its perimeter by a membranous extension applied to the surface of the substratum, offering optimal suction efficiency. Attachment is achieved with the aid of a ventral sucker formed by the modified mandibular palps and first pair of legs (Tiemann 1986). In *P. aspidosoma* the lateral margins of the cephalothorax and pleurotergites of the free pedigerous somites are fringed with closely set spinules and no specialised suction device is present. In *P. aspidosoma* the somites bearing legs 2–4 are transversally expanded forming large pleurotergites while in porcellidiids only those bearing legs 2–3 are modified in a similar way, the leg 4-bearing somite being markedly smaller (note that in members of the laophontid genus *Peltidiphonte* such well-developed pleurotergites are present on the somites bearing legs 2–5: Figure 7N). The genital complex in the Porcellidiidae forms a single pair of backwardly produced extensions which typically embrace the anal somite and caudal rami. In *P. aspidosoma* the genital double-somite has two sets of strongly developed pleurotergites and the second and third abdominal somites each one pair, the last one surrounding the anal somite and part of the caudal rami (Figure 2A).

The transition from an epibenthic to a mesopsammic life style has evolved independently and successfully many times in various lineages of the Harpacticoida. Adaptation to the three-dimensional labyrinth of the interstitial system of sand grains and shell gravel is primarily achieved by miniaturisation and/or the adoption of vermiformicity or a cylindrical body shape, thus enhancing flexibility and wriggling ability. Dwarfism often leads to a simplification in body morphology, most commonly resulting in the loss of swimming leg segments and rami or even entire limbs. Within the Laophontidae such regressive evolution linked to an interstitial mode of life can be observed in at least 14 genera characterised by a cylindrical body form, including *Laophontina* Norman & Scott, 1905, *Klieonychocamptoides* Noodt, 1958, *Afrolaophonte* Chappuis, 1960, *Stygolaophonte* Lang, 1965, *Mexicolaophonte* Cottarelli, 1977, *Galapalaophonte* Mielke, 1981, *Novolaophonte* Cottarelli, Saporito & Puccetti, 1983, *Indolaophonte* Cottarelli, Saporito & Puccetti, 1986, *Amerolaophontina* Fiers, 1991, *Wellsiphontina* Fiers, 1991, *Spiniferaphonte* Gheerardyn & Fiers, 2007, *Raowellsia* Özdikmen, 2008, *Aequinoctiella* Cottarelli, Bruno & Berera, 2008, and *Fiersiphontina* Bruno & Cottarelli, 2011 (Noodt 1958; Lang 1965; Cottarelli 1977; Mielke 1981; Cottarelli et al. 1983, 1986, 2008; Wells and Rao 1987; Fiers 1990, 1991; Gheerardyn et al. 2007; Bruno and Cottarelli 2011). An alternative – less common – adaptation to the interstitial environment is flattening of the body but this appears to occur only in copepods that inhabit substrata with larger crevices such as shell gravel. Within the Laophontidae adoption of a dorsoventrally depressed body form as an adaptation to the interstitial habitat has evolved convergently at least twice, i.e. in *Peltidiphonte* and *Philippiphonte*.

### A review of the genus *Folioquinpes* Fiers & Rutledge, 1990

Fiers and Rutledge (1990) proposed the genus *Folioquinpes* to accommodate *Laophonte chathamensis* Sars, 1905 and a new species *F. mangalis*. Sars’s (1905) description did not include a discussion on possible relationships but Sewell (1924) believed that there was a close affinity with *Laophonte mohammed* Blanchard & Richard, 1891, to the extent that both species may well turn out to be synonymous. Nicholls (1941) placed *L. chathamensis*, together with *L. mohammed* and *L. bengalensis* Sewell, 1934, in the *mohammed*-group of the genus *Laophonte*. This group of fresh and brackish water forms is effectively equivalent to the *mohammed*-group delimited by Lang (1948) within the genus *Onychocamptus* Daday, 1903. Lang (1944, 1948) resurrected the latter genus after it had previously been synonymised with *Laophonte* by Zykoff (1904). Lang did not expound on the new placement of *L. chathamensis* but it is conceivable that the short antennule in the female, the presence of only three setae on the female P5 endopodal lobe and the shape of the P1 have influenced his generic assignment.

Mielke (1981) found two ovigerous females in the Galápagos which he provisionally identified as *Onychocamptus* spec. He suspected possible conspecificity with *O. chathamensis* which was subsequently confirmed by Fiers & Rutledge (1990). The latter authors highlighted the absence of distinct swimming leg sexual dimorphism in *O. chathamensis*, a character standing in marked contrast with the modified 3-segmented P3 endopod and strongly built P2–P4 exopods exhibited by males of other *Onychocamptus* species. Sars (1905) stated that the exopods of P3–P4 were somewhat more strongly developed in the male but Fiers and Rutledge (1990) failed to find any sexual dimorphism upon re-examination of material from Papua New Guinea, Guadeloupe and the Philippines (Fiers, unpubl. data). In *F. mangalis*, they did, however, note that the outer spines on P2–P4 were stronger in the male. The foliaceous P5 exopod showing a reduced armature in the female and the absence of strongly modified P2–P4 exopods and sexual dimorphism on the P3 endopod in the male, were regarded as the primary diagnostic features of *Folioquinpes* (Fiers and Rutledge 1990).

Schizas and Shirley (1994), who were unaware of the publication of *Folioquinpes*, recognised two lineages within *Onychocamptus*, based on the shape of the P5 endopodal lobe in the female: the *mohammed*-group and the *chathamensis*-group [including *O. chathamensis* and *Onychocamptus* spec. *sensu* Mielke (1981)].

*Folioquinpes chathamensis* and *F. mangalis* assume a bizarre geographical distribution, including widely separated records from the Atlantic and Western Pacific oceanic basins. Remarkably, both species have been recorded from the northern coast of Papua New Guinea and exist in relative proximity in the Caribbean. Unless both species are widely distributed throughout the Indo-Pacific (for which there is no compelling evidence at present) this may indicate the existence of a complex of sibling species, each with a more restricted distribution. In this context, Mielke (1981) did not rule out the possibility that his Galápagos material of *F. chathamensis* represents a distinct “subspecies”.

Sewell’s (1924) specimens of *F. chathamensis* from Chilika (= Chilka) Lake, India differ from Sars’ (1905) type description in (a) the female P5, showing three outer setae on the exopod and a very short, blunt spine apically, (b) the more slender P1 endopod, and (c) the shorter P4 enp-2. Since no variability in these characters has been recorded by either Sars (1905) or Fiers and Rutledge (1990), both of whom examined ample material, the differences recorded in the Chilika Lake population are considered sufficient to warrant separate specific status; Sewell’s (1924) material is consequently renamed here as *F. indicus* sp. n. Similarly, Rühe’s (1914) record of *F. chathamensis* from South Africa requires confirmation. His illustrations show distinctly longer caudal rami, reduced pleural extensions on the abdominal somites, and longer setae on the female P5 baseoendopod. Rühe’s concise description does not enable us to reach a final verdict on the specificity of his specimens. Pending the re-examination of new material, *Laophonte chathamensis* *sensu* Rühe (1914) is here considered *species inquirenda* in *Folioquinpes*. The recent record of *F. mangalis* from South Korea (Kim 2013) is based on a misidentification and attributed below to a new species, *F. pseudomangalis* sp. n. Differentiating characters between the four species of *Folioquinpes* and those of related genera are summarised in Table 1.

**Table 1.** Number of segments in female antennule (A1), number of setae on antennary exopod (A2) and armature of P2–P4 (female) and P5 (both sexes) of members of *Onychocamptus* Daday, 1903 and allied genera.

	A1	A2	P2		P3		P4		P5♀		P5♂
			exp	enp	exp	enp	exp	enp	exp	benp	exp
***ONYCHOCAMPTUS***											
*mohammed* (Blanchard & Richard, 1891)	5	4	0.1.123	0.220	0.1.123	0.321	0.1.123	0.111	3	3	2
*bengalensis* (Sewell, 1934)	5	4	0.1.123	0.220	0.1.123	0.321	0.1.123	0.111	3	3	2
*besnardi* Jakobi, 1954	5	4	0.1.123	0.220	0.1.123	0.321^a^	0.1.022	0.111	3	3	2
*vitiospinulosa* (Shen & Tai, 1963)	5	4	0.1.123	0.220	0.1.123	0.321	0.1.123	0.111	3	2	2
*anomalus* (Ranga Reddy, 1984)	5	1	0.1.123	0.220	0.1.123	0.321	0.1.122	0.111	4	3	3
*taifensis* Kikuchi, Dai & Itô, 1993	5	4	0.1.123	0.120	0.1.123	0.321	0.1.123	0.111	3	3	2
*krusensterni* Schizas & Shirley, 1994	5	4	0.1.123	0.220	0.1.123	0.321	0.1.122^b^	0.111	3	3	2
***FOLIOQUINPES***											
*chathamensis* (Sars, 1905)	5	4	0.1.123	0.220	0.1.123	0.321	0.1.123	0.111	3	3	2^c^
*indicus* **sp. n.**	5	4	0.1.123	0.220	0.1.123	0.321	0.1.123	0.111	4	3	?
*mangalis* Fiers & Rutledge, 1990	4	4	0.1.123	0.220	0.0-1^d^.123	0.221	0.0.123	0.111	4	2	2
*pseudomangalis* **sp. n.**	4	4	0.1.123	0.220	0.1.123	0.221	0.0.123	0.111	4	3	2
***ONYCHOQUINPES***											
*permixtionis* Gómez & Morales-Serna, 2013	5	4	0.1.123	0.220	0.1.123	0.321	0.1.123	0.111	3	3	2
***PSAMMOLAOPHONTE*^e^**											
*spinicauda* Wells, 1967	5	2	0.0.022	0.020	0.0.022	0.021	0.0.022	0.011	4	3	3

^a^: corrected by Lee and Huys (1999: 318); ^b^: female condition (0.1.123 in male); ^c^: see text for reinterpretation; ^d^: 0 = typical condition; ^e^: The presence of a 5-segmented ♀ antennule, sensillar tubercles (socles) on the body somites, a strongly developed seta V on the caudal ramus and a trisetose ♀ P5 baseoendopod in *P. spinicauda* (cf. Wells 1967) indicates that this genus is closely affiliated to the *Onychocamptus*-*Folioquinpes*-*Onychoquinpes*-group of genera.

**Diagnosis.** Laophontidae. Body moderately to strongly dorsoventrally depressed. Integument of cephalothorax and body somites with dense pattern of long spinules; dorsal posterior margins of somites with sensillate tubercles. Rostrum partially delimited at base; prominent and bell-shaped, with (*F. mangalis*, *F. pseudomangalis* sp. n.) or without (*F. chathamensis*, *F. indicus* sp. n.) spinules between apical sensilla. Genital double-somite ♀ bilaterally incised, with dorsal and lateral transverse chitinous ribs marking original segmentation. Pleural extensions of ♀ abdominal somites moderately to strongly (conical) developed. Caudal ramus elongate, cylindrical, with spinules along inner and (often) outer margin; with seven setae; seta V well developed, with fracture plane, fused to short seta IV; seta VI reduced, setiform; ramus slightly sexually dimorphic in *F. mangalis* (inner margin less convex in ♂). Anal operculum spinulose.

Sexual dimorphism in antennule, P5, P6 and in genital segmentation. Slight dimorphism in exopods of P3–P4, abdominal ornamentation and caudal ramus shape.

Antennule short and 4- or 5-segmented in ♀, all segments densely spinulose; 8-segmented and subchirocer with three segments distal to geniculation in ♂; segment 1 with strong spinules along anterior margin; segment 2 sometimes with small blunt process near posterior margin; with aesthetasc on segment 3 (♀) or 5 (♂) and probably as part of acrothek on apical segment; segment 6 ♂ with three hyaline extensions. Antenna with four setae on exopod; allobasis with abexopodal seta. Mandibular palp elongate, 1-segmented; with one basal, one exopodal and three endopodal setae. Maxillule with defined exopod bearing two setae. Maxilla with three endites on syncoxa; endopod represented by two setae. Maxilliped moderately robust; syncoxa with one seta; basis with spinules along both margins; endopodal claw curved, with accessory seta at base.

P1 with 2-segmented exopod, with long pinnate outer spine on exp-1, and three spines and two geniculate setae on exp-2; endopod moderately stout, enp-1 without inner seta, enp-2 with minute seta and short, strong claw. Swimming legs with 3-segmented exopods and 2-segmented endopods in both sexes; segments and/or outer spines of P3–P4 exopods somewhat stronger in ♂. Armature formula as follows:

**Table:** 

	Exopod	Endopod
P2	0.1.123	0.220
P3	0.(0–1)*.123*: variability in *F. mangalis*	0.(2–3)21
P4	0.(0–1).123	0.111

P5 ♀ large, with separate, densely hirsute rami; exopod elongate-oval, arising from pedestal, with 2–3 setae laterally and one short dilated spine apically; baseoendopod with triangular or rectangular endopodal lobe bearing one apical and 1–3 lateral setae. P5 ♂ incorporated into supporting somite; endopodal lobe completely absent (no armature); exopod typically not defined at base, small, with two setae.

P6 ♀ with two minute setae; P6 ♂ asymmetrical, membranous flaps with two setae.

Copepodids IV–V without modified P4 in ♀ (*cf.* Fiers 1998).

Euryhaline but primarily brackish or freshwater, free-living.

**Type species.** *Folioquinpes mangalis* Fiers & Rutledge, 1990 (by original designation).

**Other species.** *F. chathamensis* (Sars, 1905), *F. indicus* sp. n., *F. pseudomangalis* sp. n.

**Species inquirenda.** *Folioquinpes chathamensis* (Sars, 1905) *sensu* Rühe (1914)

### Key to species

**Table:** 

1	Anterior margin of rostrum with spinules between sensilla; antennule ♀ 4-segmented; P3 enp-2 with two inner setae; P3–P4 exp-2 without inner seta	**2**
–	Anterior margin of rostrum without spinules between sensilla; antennule ♀ 5-segmented; P3 enp-2 with three inner setae; P3–P4 exp-2 with inner seta	**3**
2	Cephalothorax bilaterally incised; P5 ♀ endopodal lobe with two setae	***F. mangalis* Fiers & Rutledge, 1990**
–	Cephalothorax not bilaterally incised; P5 ♀ endopodal lobe with three setae	***F. pseudomangalis* sp. n.**
3.	P4 enp-2 longer than enp-1; P5 exopod ♀ with two outer setae	***F. chathamensis* (Sars, 1905)**
–	P4 enp-2 shorter than enp-1; P5 exopod ♀ with three outer setae	***F. indicus* sp. n .**

#### Folioquinpes chathamensis

Taxon classificationAnimaliaHarpacticoidaLaophontidae

(Sars, 1905)

Laophonte chathamensis Sars, 1905
Folioquinpes chathamensis (Sars, 1905) Fiers and Rutledge (1990)
Onychocamptus spec. *sensu*Mielke (1981): Fiers and Rutledge (1990)

##### Original description.

Sars (1905): 391–393; Plate 17 (figs 103–118).

##### Additional description.

Mielke (1981 as *Onychocamptus* spec.): 52; Abb. 28.

##### Type locality.

New Zealand, Chatham Islands, Wharekauri (= Chatham Island), Te Whanga Lagoon; shallow brackish water.

##### Body length.

480 μm (♀), slightly smaller (♂) [Sars 1905]; 430–450 μm (♀) [Mielke 1981].

##### Remarks.

Fiers and Rutledge (1990) stated that armature and shape of the male P5 differed between *F. chathamensis* and *F. mangalis*. Sars’s (1905) text description is not informative with regard to the number and position of armature elements. His figure (figure 118) suggests that the P5 is distinctly bilobate, having one endopodal and three exopodal setae. However, the accompanying figure legend states that the left member is illustrated, implying that Sars had figured it in dorsal aspect. The “endopodal” seta is therefore the outer basal arising from a setophore (and not an endopodal lobe). Comparison with *F. mangalis* also suggests that there are only two exopodal elements, the third one representing the sensilla originating from a lateral tubercle. Based on this reinterpretation there is probably no difference in male P5 morphology between both species. The absence of the typical baseoendopodal incision in the female P5, separating the endopodal lobe and the pedestal bearing the exopod, is also attributable to an observational error by Sars (1905: Taf. 17, fig. 116).

*Folioquinpes chathamensis* resembles *F. indicus* sp. n. in the absence of spinules along the anterior margin of the rostrum, the 5-segmented condition of the female antennule, the presence of three inner setae on the distal endopodal segment of leg 3, and of the inner seta on the middle exopodal segment of legs 3–4. The alternative states, including the 4-segmented female antennule, are displayed in the other two species of the genus (Table 1).

Hamond (in Hicks 1977a: 457) collected *F. chathamensis* near Sydney and Melbourne while Newton and Mitchell (1999) obtained it in mud samples from the Hopkins River estuary in south-western Victoria. It remains unclear whether Lewis’s (1984) single record from an estuarine lagoon in New Zealand is new or refers to Sars’s (1905) type locality. Fiers (1995) recorded the species from the ‘aufwuchs’ covering submerged mangrove pneumatophores in the Celestún Lagoon, northwest of the Yucatán Peninsula (Mexico). Gómez and Morales-Serna (2013) erroneously cited Suárez-Morales et al. (2009) as the source for the Gulf of Mexico record but their checklist only refers to Fiers and Rutledge’s (1990) record of *F. mangalis* from Louisiana. The latter authors also examined material from Guadeloupe, Papua New Guinea and Taal (Bombón) Lake, a freshwater lake on the island of Luzon in the Philippines (Fiers, unpubl. data). Mielke (1981, 2003) found the species in a sandy beach in Bahía Academy (Santa Cruz), Galápagos. A single African outlier has been reported from the brackish coastal Ebrié Lagoon in Ivory Coast (Dumont and Maas 1988). The records by Rühe (1914) and Sewell (1924) refer to other species (see below).

Newton and Mitchell (1999) observed during estuarine mud incubation experiments that *F. chathamensis* developed to egg-bearing female stage in only six days at 20°C, suggesting that dormancy occurred at an advanced copepodid stage rather than the egg.

#### Folioquinpes mangalis

Taxon classificationAnimaliaHarpacticoidaLaophontidae

Fiers & Rutledge, 1990

##### Original description.

Fiers and Rutledge (1990): 122–124; fig. 9.

##### Type locality.

Papua New Guinea, Capital District, Motupore Island; mangrove along northern shore; algae on pneumatophores.

##### Body length.

600 μm (♀), 400 μm (♂) [Fiers and Rutledge 1990].

##### Remarks.

*Folioquinpes mangalis* differs from its congeners in the bilaterally incised cephalothorax and the more strongly developed P5 ♀ endopodal lobe which bears only two setae. The dense spinular ornamentation on the anterior surface of leg 5 has not been documented in other species of the genus. The species is similar to *P. pseudomangalis* sp. n. in the strongly depressed body, the distinct pleural extensions on the urosomites, the 4-segmented female antennule, the lack of the inner seta on P4 exp-2 (and P3 exp-2 but see below) and the presence of only two inner setae on P3 enp-2.

Fiers and Rutledge (1990) found two specimens with an inner seta on P3 exp-2; the absence of this seta appears to represent the normal condition. They also figured only two outer spines on P1 exp-2 (their figure 9g) but mentioned three in the text, which is here regarded as the correct condition.

*Folioquinpes mangalis* has been found on pneumatophores of mangrove trees along the southern (type locality) and northern coast (Sepik River delta) of Papua New Guinea and on *Spartina alterniflora* stems from marshes in Cocodrie, Louisiana (Fiers and Rutledge 1990; Rutledge and Fleeger 1993). It was subsequently found in samples of decaying leaves and sediment, from a *Rhizophora apiculata*-dominated mangrove forest bordering the Sungai Merbok estuary in north-western peninsular Malaysia (Gee and Somerfield 1997; Somerfield et al. 1998). Kim (2013) recently identified two specimens from Jeju Island, Korea as *F. mangalis* but this material is believed to represent a different species (see below).

#### Folioquinpes indicus sp. n.

Taxon classificationAnimaliaHarpacticoidaLaophontidae

http://zoobank.org/DB1E5425-B537-4C6D-8F79-DD695B7C7B75

Laophonte chathamensis Sars, 1905 *sensu*Sewell (1924)

##### Original description.

Sewell (1924 as *Laophonte chathamensis*): 830–832; Plate LVII, fig. 2 (♀ only).

##### Type material.

The original material collected by R.B. Seymour Sewell is no longer available for re-examination. In accordance with ICZN (1999) Arts 16.4 and 72.5.6 the female specimen illustrated by Sewell (1924) in his plate LVII (fig. 2) is here fixed as the holotype of *F. indicus* sp. n.

##### Type locality.

India, Odisha State, Chilika (Chilka) Lake; anchorage at Barkul due east; tow-nettings of brackish water plankton.

##### Body length.

400 μm (♀) [Sewell 1924].

##### Remarks.

Females of *F. indicus* differ from those of *F. chathamensis* primarily in the morphology of the P5 exopod which is more oval, has three outer setae (instead of two) and a very short, blunt spine apically (Sewell may have missed the flagellate tip). Additional differences include the more slender P1 endopod (enp-1:enp-2 ratio 5.3 *vs* 4.6) and the shorter P4 enp-2 (enp-1:enp-2 ratio 1.1 *vs* 0.8).

The authenticity of other records from the Indian peninsula is unclear since none was accompanied by illustrations. Chappuis (1941) recorded *Onychocamptus chathamensis* from the River Sina and the River Bhima (near Pandharpur) in Maharashtra State, approximately 250 km inland from the Indian west coast. In a later report Chappuis (1954) added records from Mhaisgaon (River Sina) and Dabhol (Vashishti River), both in Maharashtra State, and from coastal lagoons in two districts of the Union Territory of Puducherry, *i.e.* Mayyazhi (Mahé) and Karaikal, along the southwestern and southeastern coasts of the Indian peninsula, respectively. *Folioquinpes chathamensis* has recently been recorded from the middle and/or lower reaches of the River Godavari and River Krishna in Andhra Pradesh (Jayaram 1995; Ranga Reddy 2001, 2014; Ranga Reddy and Schminke 2009a–b; Ranga Reddy and Totakura 2010; Totakura et al. 2016). These hyporheic freshwater records, all from the east coast of India, most likely refer to *F. indicus*. Ranga Reddy (2002) reported “*F. chathamensis*” from a bore well on the Nagarjuna University campus, near Guntur town (Andhra Pradesh). The species is also known from Port Canning near Kolkata, West Bengal (Forró and Dussart 1985).

#### Folioquinpes pseudomangalis sp. n.

Taxon classificationAnimaliaHarpacticoidaLaophontidae

http://zoobank.org/37483F03-31C2-4710-9180-DD05779A6440

Folioquinpes mangalis Fiers & Rutledge, 1990 *sensu*Kim (2013)

##### Original description.

Kim (2013 – as *Folioquinpes mangalis*): 38–43; figs 13–16.

##### Type locality.

Korea, Jeju Island, Aewol; washings of invertebrates and intertidal stones.

##### Type material.

In accordance with ICZN (1999) Arts 16.4 and 72.5.6 the female specimen illustrated by Kim (2013) in his fig. 13A is here fixed as the holotype of *F. pseudomangalis* sp. n.

##### Body length.

600 μm (♀), 400 μm (♂) [to be confirmed – see below].

##### Remarks.

Kim (2013) copied Fiers and Rutledge’s (1990) text description virtually *verbatim* (with the exception of the mouthparts which were not described in the original account). This explains the discrepancies between Kim’s (2013) text and some of his illustrations and also casts doubt on the accuracy of the body length given for both sexes of the Korean specimens which is allegedly identical to that of *F. mangalis*.

Kim’s (2013) specimens are most similar to *F. mangalis* but differ from Fiers and Rutledge’s description in a number of characteristics, justifying their assignment to a distinct species: (a) cephalothorax not bilaterally incised, (b) caudal rami relatively shorter, (c) second antennulary segment ♀ without blunt process, (d) both exopod and endopod of P4 markedly less elongate, (e) ♀ P5 endopodal lobe with three setae and markedly shorter while exopod relatively more slender, and (f) ♀ P5 rami without dense spinular ornamentation on anterior surface.

#### Folioquinpes chathamensis

Taxon classificationAnimaliaHarpacticoidaLaophontidae

(Sars, 1905) sensu Rühe (1914)

Laophonte chathamensis Sars, 1905 *sensu*Rühe (1914)

##### Original description.

Rühe (1914): 33; fig. 11 (♀ only).

##### Type locality.

South Africa, Western Cape Province, Cape Town, Muizenberg, Sandvlei; freshwater lake.

##### Body length.

470–670 μm (♀) [Rühe 1914].

##### Remarks.

Rühe’s (1914) illustrations are limited to the P5 and the abdomen in dorsal aspect. Differences with *F. chathamensis* include the distinctly longer caudal rami, the reduced pleural extensions on the abdominal somites, and the setae on the P5 baseoendopod being distinctly longer. Rühe (1914) suspected that Sars (1905) had misinterpreted the apical blunt spine on the P5 exopod as a single element rather than two adjacent ones. Mielke’s (1981) illustration, which confirms Sars’s observation, indicates that the space between the two apical spines in Rühe’s fig. 11b is in reality the inner core of the basally dilated spine. We suspect that Rühe has misinterpreted as a real phenomenon what he has seen only in optical section. Pending the discovery of fresh material the Western Cape population attributed to *Folioquinpes chathamensis* is here regarded as a *species inquirenda* in the genus.

## Supplementary Material

XML Treatment for Philippiphonte

XML Treatment for Philippiphonte aspidosoma

XML Treatment for Folioquinpes chathamensis

XML Treatment for Folioquinpes mangalis

XML Treatment for Folioquinpes indicus

XML Treatment for Folioquinpes pseudomangalis

XML Treatment for Folioquinpes chathamensis
